# Marine and Coastal Science: Where Space and Ocean Meet

**DOI:** 10.1289/ehp.115-a243a

**Published:** 2007-05

**Authors:** David C. Holzman

A variety of oceanborne microbes can sicken humans via seafood, drinking water, and swimming. At the February 2007 annual meeting of the American Association for the Advancement of Science, experts described new technologies for predicting hidden or nascent oceanborne hazards, often by satellite. Such predictions could prevent illness without causing economic losses that might otherwise occur—for example, when shellfish beds are shut down needlessly.

Location, time, and intensity of cholera epidemics can now be accurately predicted from satellite observations of sea surface temperature, sea surface height, and chlorophyll in the water, said Rita R. Colwell, a distinguished university professor at the University of Maryland. The correlations of disease with these environmental conditions are logical. Sea surface temperature and sunlight nurture phytoplankton, the food source for the copepods that carry *Vibrio cholerae*. Chlorophyll in the water indicates the presence of phytoplankton. Sea surface elevation is a proxy for the tides, which influence estuarine salinity; a high sea surface means pathogens can be washed into estuaries.

With enough warning, even people in the least stable situations—for example, in war-torn developing countries—could filter drinking water using low-tech devices, cutting the infection rate by 50%, said Colwell. She was the first to monitor and predict cholera epidemics in Bangladesh using satellites, as reported in the 15 February 2000 issue of *Proceedings of the National Academy of Sciences*.

Similarly, D. Jay Grimes, provost and vice president for academic affairs at the University of Southern Mississippi, is modeling prediction of health hazards from *V. parahaemolyticus* and *V. vulnificus* in the Gulf of Mexico and off the East Coast. In seawater or undercooked seafood these organisms can cause acute gastroenteritis and septicemia. *V. vulnificus* also infects wounds. Mortality is estimated at about 50–60% in compromised patients.

Grimes envisions a national surveillance system based on his model, which would integrate satellite measurements of sea surface temperature, chlorophyll, salinity, and turbidity. The multiplicity of parameters is needed because, for example, whereas temperature correlates well with the presence of the two microbes, it doesn’t distinguish between pathogenic and nonpathogenic *Vibrio* species. However, turbidity does appear to do so.

Many more potentially toxic species thrive in algal blooms. Two dozen such organisms are known to frequent the waters off the South Carolina coast, says Patrick J.P. Brown, a research specialist at the Hollings Marine Laboratory. Identifying which are in any given water sample at any given point in time is costly and time-consuming. The tab for analyzing one sample for 11 species using real-time polymerase chain reaction is about $650, and microscope identification requires extensive training of personnel.

Brown has developed a highly sensitive DNA amplification technique he calls “species identity via chimeric amplification” (SIVCA) that can assay 11 species in a water sample for $60. The technique has been successfully tested in the field, he says. This new tool will enable resource managers to make decisions on beach and shellfish bed closings and advisories much more quickly than current methods do.

Old-fashioned observation underpins high-tech efforts to anticipate domoic acid contamination in shellfish in the Pacific Northwest. This algal toxin can cause short-term memory loss, brain damage, and death. Recent research cruises established that the biggest blooms with the highest toxin levels are associated with offshore eddies or upwelling zones near coastal promontories, and that storm conditions tend to push the toxin-producing algae coastward.

Vera L. Trainer, program manager for the Harmful Algal Blooms Program at the Northwest Fisheries Science Center, has proposed placing early-warning moorings at these locations. These environmental sensing platforms are designed to record the presence and number of microorganisms and toxins using genetic and immunological techniques, respectively. This information could be integrated with data from various sensors and satellite images to serve as a reliable early warning for harmful algal blooms.

As the session concluded, Michael Moore, a senior research specialist at the Woods Hole Oceanographic Institution, noted that researchers have only begun to assess the scope of the hazards in oceans. Moore is documenting the diversity and prevalence of zoonotic organisms in marine mammals and seabirds. So far, in 116 individual birds and mammals, he has detected 79 of more than 200 known zoonotic bacteria capable of causing human disease. He was the first to detect a *Brucella*-like agent in birds and has also found moderate to high antibiotic resistance in bacteria from marine birds and animals.

